# Inferior alveolar nerve injury following calcium hydroxide extrusion: a case report with preventive insights and three-year follow-up

**DOI:** 10.3389/fdmed.2026.1794033

**Published:** 2026-03-25

**Authors:** Sofia Drouri, Chaimae Ghouni, Khaoula Sadel, Hafsa El Merini

**Affiliations:** Department of Conservative Dentistry and Endodontics, Faculty of Dental Medicine, Hassan II University, Casablanca, Morocco

**Keywords:** accidental extrusion, barium sulphate, calcium hydroxide, endodontic therapy, inferior alveolar nerve

## Abstract

**Background:**

Calcium hydroxide is commonly used in endodontics for its antimicrobial properties. However, accidental extrusion beyond the apex can cause severe complications, including injury to the inferior alveolar nerve (IAN), which provides sensory innervation to the lower lip, chin, and posterior mandibular region.

**Case presentation:**

A 35-year-old female undergoing root canal treatment of tooth 46 developed paresthesia of the lower lip and chin due to accidental extrusion of calcium hydroxide along the IAN canal, confirmed on initial cone-beam computed tomography (CBCT). Immediate management included anti-inflammatory therapy and vitamin B12 supplementation.

**Outcome:**

Over a three-year follow-up, symptoms markedly improved with partial sensory recovery and attenuation of hyperesthesia. Follow-up CBCT showed resorption of the extruded calcium hydroxide compared to the initial radiograph.

**Conclusion:**

This case highlights the critical importance of precise working length determination, controlled intracanal medicament delivery, and thorough anatomical knowledge of the IAN to prevent iatrogenic injuries during endodontic procedures.

## Introduction

Calcium hydroxide (Ca(OH)_2_), a mainstay intracanal medicament in endodontics, delivers antiseptic, antibacterial, and anti-inflammatory effects through its high pH (12.5–13). It disinfects root canals, neutralizes bacterial endotoxins, and promotes periapical healing—making it ideal for persistent infections, apical periodontitis, or multivisit treatments. However, accidental extrusion beyond the apical foramen can lead to serious complications, including injury to the inferior alveolar nerve, which provides sensory innervation to the lower lip, chin, and posterior mandibular region (1–4).

To date, the adverse effects associated with calcium hydroxide extrusion have been reported in a relatively limited number of case reports and case series (3).

The aim of this article is to present a case of inferior alveolar nerve paresthesia following the extrusion of a calcium hydroxide-based paste during endodontic therapy, and to offer recommendations for preventing such iatrogenic complications.

## Case report

This case report is described following the requirements of Preferred Reporting Items for Case Reports in Endodontics (PRICE). The patient gave written informed consent for all procedures and the publication of this report.

**Figure 1:**
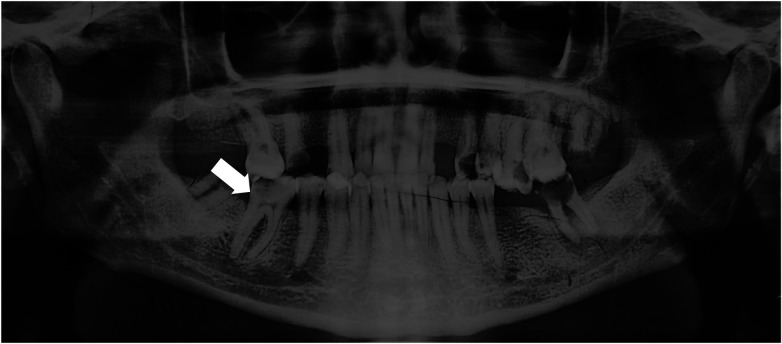
Preoperative panoramic radiograph showing a coronal radiolucency extending to the inner third of dentin in tooth 46. Note the close anatomical relationship between the roots and the inferior alveolar nerve canal (distance <4 mm).

A 35-year-old female patient presented to the Emergency department, Ibn Rochd dental consultation and treatment center of Casablanca, with spontaneous pain localized to the mandibular right first molar (tooth 46). The patient reported being in good general health, with no history of systemic or neurological disease, no prior surgeries or hospitalizations, no ongoing medications, and no known drug allergies. Clinical examination revealed an exaggerated and lingering response to cold pulp vitality testing on tooth 46, with pain persisting for approximately eight seconds after removal of the stimulus. The tooth was tender to vertical percussion, with no tenderness to palpation in the surrounding tissues. Radiographic examination (Figure 1), limited to a panoramic radiograph obtained during the emergency visit due to the absence of a preoperative periapical radiograph, revealed an extensive carious lesion involving the pulp chamber of tooth 46. Based on the clinical and radiographic findings, a diagnosis of symptomatic irreversible pulpitis associated with symptomatic apical periodontitis was established.

Endodontic treatment was promptly initiated, followed by an inter-appointment intracanal application of injectable calcium hydroxide (Ultracal XS). Overfilling of the material beyond the apex occurred inadvertently due to intracanal pressure.

Shortly after intracanal placement of the medicament, the patient reported the onset of paresthesia affecting the right lower lip, cheek, and gingival region of the right mandible.

**Figure 2:**
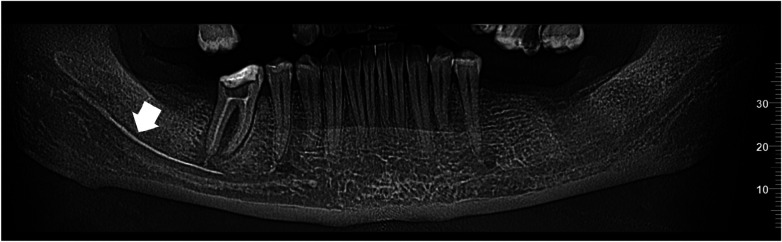
Panoramic CBCT scan showed hyperdense material emanating apically from tooth 46 extending along the mandibular canal.

**Figure 3:**
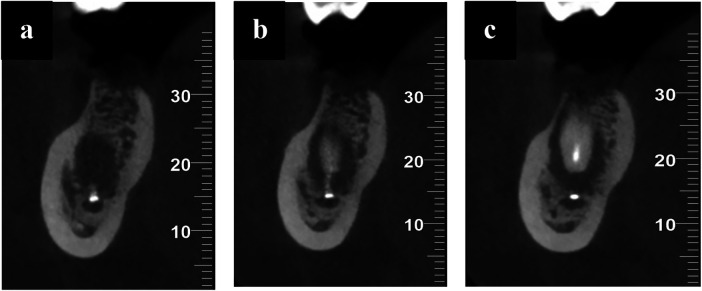
**(a–c)** sagittal sections from the CBCT scan showing the presence of radiopaque material in the periapical region of tooth 46, extending around and into the right mandibular canal following extrusion of calcium hydroxide.

The patient was subsequently referred to the Department of Conservative Dentistry and Endodontics for evaluation and further management of the complication. Cone-beam computed tomography (CBCT) (Figures 2, 3) revealed significant extrusion of calcium hydroxide into the mandibular canal, extending from the periapical area of tooth 46 toward the right mandibular ramus.

The patient was informed about the iatrogenic complication, likely due to the extrusion of Ultracal XS, resulting in injury to the inferior alveolar nerve. Medical management included administration of a vitamin B complex: Citoneurin (a combination of thiamine nitrate, cyanocobalamin, and pyridoxine hydrochloride) at a dose of 1.5 mg/day for one month, and systemic corticosteroid therapy (Prednisone 1 mg/kg/day) to reduce inflammation and promote nerve recovery.

**Figure 4:**
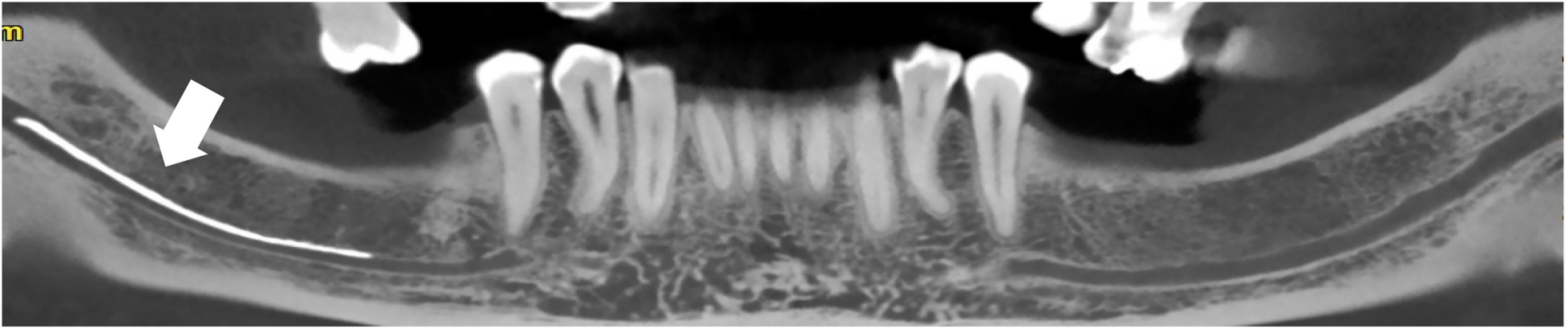
Coronal CBCT section taken after 2 years of follow-up revealed bone healing following extraction of the tooth 46, with persistence of the material in the IAN canal and slight resorption.

**Figure 5:**
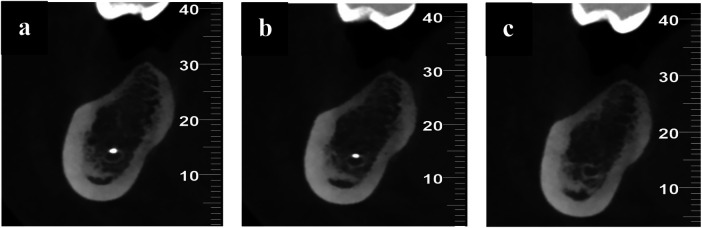
**(a–c)** sagittal CBCT sections taken after two years of follow-up, showing IAN canal with incomplete canal obturation.

**Figure 6:**
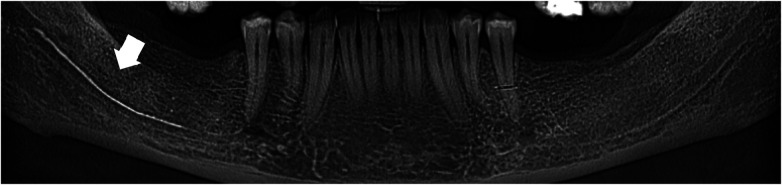
Coronal CBCT section taken after 3 years of follow-up revealed bone healing following extraction of the tooth 46, with persistence of the material in the IAN canal and partiel resorption.

**Figure 7:**
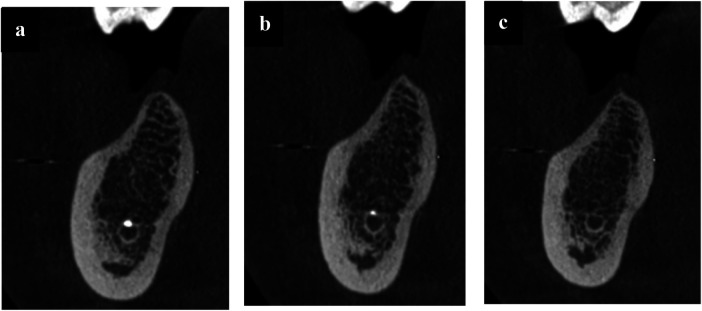
**(a–c)** sagittal CBCT sections taken after three years of follow-up, showing IAN canal with incomplete canal obturation.

After a detailed explanation of the clinical situation and available treatment options, tooth 46 was extracted at the patient's request. Long-term neurological follow-up was then initiated. Two-year (Figures 4, 5) and three-year (Figures 6, 7) follow-up assessments demonstrated partial recovery of sensory function, as well as a reduction in hyperesthesia. A follow- CBCT revealed partial resorption of the extruded calcium hydroxide compared to the initial radiograph.

## Discussion

This case report describes an iatrogenic complication during endodontic treatment caused by the overextension of calcium hydroxide into the inferior mandibular canal, leading to acute pain and paresthesia of the right lower lip.

CBCT studies have quantitatively assessed the anatomical relationship between mandibular molar apices and the inferior alveolar canal (IAC), reporting mean apex–canal distances of approximately 4–5 mm for first molars, with progressively smaller values for second and third molars (5–7). Recent investigations have shown that, in a considerable proportion of posterior mandibular teeth, the IAN is located within 3 mm of the anatomical apex (6). Based on these CBCT measurements, an apex–canal distance of ≤3–4 mm can reasonably be considered a ‘close’ anatomical relationship, reflecting a higher-risk proximity zone (7, 8).

Although rare, the extrusion of calcium hydroxide highlights a serious neurological emergency that requires prompt diagnosis and management to prevent permanent damage. Treatment should aim to reassure the patient, relieve symptoms, and promote nerve healing (4, 9).

### Pathophysiology and composition-related toxicity

Calcium hydroxide paste typically consists of calcium hydroxide powder combined with a vehicle (aqueous, viscous, or oily) and a radiopacifier such as barium sulfate (BaSO₄) to allow visualization of root canal filling (10).

Ultracal XS contains approximately 35%–40% calcium hydroxide, 30%–35% BaSO₄, and 25%–35% vehicles and excipients. Each component can contribute to tissue injury: calcium hydroxide may induce tissue necrosis and neural cytotoxicity; BaSO₄ may provoke mechanical irritation; vehicle can facilitate rapid tissue diffusion (10).

Calcium hydroxide is poorly soluble at body temperature and may persist in soft tissues. An *in vitro* study by Serper et al. (11) demonstrated that calcium hydroxide can induce nerve inflammation, foreign body reactions, and bone necrosis as it's highly caustic (pH ≈ 12.5). Furthermore, prolonged exposure of nerve tissue -beyond 30 min- may lead to irreversible conduction block, likely due to excessive amounts of calcium hydroxide disrupting the nerve cell membrane potential (2).

Also, the type of vehicle (aqueous, viscous, or oily) affects the rate and distance of diffusion. Aqueous formulations (e.g., Ultracal XS) allow faster ionic release and greater dispersion through tissues, increasing the risk of spreading into periapical spaces or neurovascular canals.

### Etiology of extrusion

Several predisposing factors have been identified in the accidental extrusion of calcium hydroxide beyond the apical foramen (11–13). These include:

- The use of low-viscosity pastes combined with high-pressure injection systems;
- Open-ended needles without lateral vents;
- Apical over-enlargement or resorption;
- Immature root apices and lack of apical constriction;
- Operator-related factors such as inadequate tactile control, absence of an apical stop, and reliance on mechanical delivery rather than manual placement (14, 15).

The extent of extrusion depends on multiple parameters: paste viscosity, syringe design, needle diameter and insertion depth, as well as the final apical preparation size and taper. Excessive pressure or blockage within the delivery needle can further increase intracanal pressure, forcing the paste beyond the apex (14).

### Clinical management and prognosis

Immediate and meticulous management of calcium hydroxide extrusion is essential, as it represents a true neurological emergency. The management of this endodontic complication remains controversial, varying from a wait-and-see approach, including anti-inflammatory drugs and periodic follow-up, to early, if not immediate, surgical intervention, including debridement of the mandibular canal and decompression of the inferior alveolar nerve.

Initial management focused on conservative measures: copious irrigation of the root canal system with sterile saline to promote bleeding and drainage of contaminated tissues. In cases of severe pain, supplemental anesthesia -preferably via nerve block- should be administered. Throughout the process, clear communication and reassurance are essential to reduce anxiety and enhance cooperation (14, 16, 17).

Medical management typically includes appropriate analgesics, corticosteroids at a dosage of 1 mg/kg administered as a single morning dose for four days, and a B-complex vitamin regimen for two to four weeks depending on the severity of neurological involvement. Cold compresses are recommended during the first 24 h to reduce inflammation, followed by warm compresses to promote local circulation (17, 18).

If neurological symptoms persist beyond 24–72 h, referral to a specialist is essential. In severe cases, surgical removal of the extruded material or even dental extraction may be necessary to prevent irreversible nerve damage (13, 19). Hence, when acute and persistent pain develops fol lowing accidental apical extrusion of great amounts of Ca(OH)_2_ paste, conservative surgical treatment including tooth extraction following by socket curettage may be recommended to relief mechanical compression of the peri apical tissues, promptly controlling pain and preventing necrosis of adjacent soft tissue and bone (10). In the present case, the patient refused surgical debridement of the inferior alveolar canal and decompression of the inferior alveolar nerve and opted instead for extraction of the causal tooth.

Severe pain following extrusion of endodontic materials requires early diagnosis and prompt management to minimize the risk of permanent nerve injury. Cone-beam computed tomography (CBCT) can be particularly valuable in assessing the spatial relationship between the extruded material, the mandibular canal, and surrounding bone, allowing better monitoring of tissue response and possible resolution.

### Prevention and clinical recommendations

To prevent such complications, strict adherence to technical protocols is essential. Preoperative radiographs should be carefully analyzed to assess the proximity of the tooth to critical anatomical structures-particularly in high-risk regions such as mandibular premolars and molars. The indication for inter-appointment dressing should be justified, and the method of application individualized.

Over-instrumentation must be avoided, especially in anatomically delicate areas. When applying calcium hydroxide, it is advisable to use custom-prepared pastes, delivered via a lentulo spiral or paper points to enable better control. If syringe delivery is used, the manufacturer's instructions must be followed precisely. The diameter and needle insertion depth should be adapted to the canal's taper and diameter to minimize the risk of extrusion. Injection must be slow and progressive, with the needle withdrawn gradually and without applying excessive pressure. Postoperative radiographs are essential to verify that no material has been extruded beyond the apex (14–16).

Although extrusion of calcium hydroxide cannot always be completely prevented, early recognition of inadvertent extrusion and limitation of the extruded volume are clinically achievable objectives. The use of magnification devices such as dental operating microscopes or magnification loupes may help clinicians visually assess intracanal conditions during medicament placement.

In addition, commercially available injectable calcium hydroxide syringes with calibrated delivery systems may help control the amount of medicament introduced into the canal, thereby reducing the risk of excessive extrusion. Products providing volume guidance per canal may offer an additional safety measure during intracanal medicament placement.

Finally, this case underscores the need for updated clinical guidelines specifically addressing the management of intracanal medicament extrusions, as current recommendations rely primarily on individual case experiences.

### Study limitations

As a single-case report, the findings cannot be generalized or used to draw broad conclusions regarding the risks of Ca(OH)₂ extrusion or associated recovery patterns. Similar published cases highlight both the rarity and variability of such events, underscoring the need for larger cohort studies to better quantify incidence and identify potential predictors, such as root proximity to the inferior alveolar nerve (IAN) canal. In this case, the absence of a preoperative CBCT limited the anatomical assessment; however, careful interpretation of available periapical radiographs remains essential, and CBCT could provide additional information if available.

## Conclusion

Accidental extrusion of calcium hydroxide into the mandibular canal is a rare but serious complication that may result in severe pain and long-term neurological damage.

Its management requires immediate recognition, careful clinical evaluation, and appropriate intervention to minimize permanent nerve injury. Prevention remains the best approach-through precise working length control, gentle application techniques, and awareness of anatomical proximity to critical structures.

The use of CBCT can aid in accurate assessment and follow-up, while early pharmacologic management with corticosteroids, analgesics, and neuroprotective agents may enhance recovery.

## Data Availability

The original contributions presented in the study are included in the article/Supplementary Material, further inquiries can be directed to the corresponding author.
